# Altered cardiac contractility and aerobic muscular capacity markers during exercise in patients with obesity and DMT II

**DOI:** 10.1186/s13102-025-01145-y

**Published:** 2025-04-28

**Authors:** Stefan Kwast, Johannes Lässing, Roberto Falz, Jana Hoffmann, Christoph Pökel, Antina Schulze, Thomas Schröter, Michael Borger, Martin Busse

**Affiliations:** 1 Institute of Sports Medicine and Prevention, University Leipzig, Rosa-Luxemburg-Str. 30, 04103 Leipzig, Germany; 2 Department of Exercise Science and Sports Medicine, University Halle-Wittenberg, Von-Seckendorff-Platz 2, 06120 Halle (Saale), Germany; 3 University Leipzig, Sports Medicine Outpatient Clinic, Rosa-Luxemburg-Str. 30, 04103 Leipzig, Germany; 4 Leipzig Heart Center, Department of Cardiac Surgery, University Leipzig, Strümpelstr. 39, 04289 Leipzig, Germany

**Keywords:** Cardiac power output, Diabetes, Obesity, Exercise performance, Oxygen consumption, Stroke work, Muscle

## Abstract

**Background:**

Impaired exercise capacity influences obesity and diabetes disease progression and vice versa. The primary objective of this prospective, observational, real-world study was to characterize exercise capacity in patients with obesity or type II diabetes mellitus and healthy controls by cardiac capacity (cardiac output (CO), cardiac power output (CPO)) and peripheral muscle capacity (peak power output (Pmax) and arterio-venous oxygen difference (avDO2)). The effects of an exercise and lifestyle intervention on these cardiac and peripheral muscular markers in obese and diabetic patient groups were additionally evaluated.

**Methods:**

At a university sports medicine outpatient clinic, 24 obese (OB) and 38 diabetes mellitus type II (DM) patients and 20 healthy controls (HE) were investigated in a cross-sectional analysis. OB and DM were reexamined after a standard of care exercise intervention. Parameters were assessed at rest and during a cardiopulmonary exercise test (CPET). Blood pressure, impedance cardiography, and respiratory gas analysis were continuously recorded during CPET.

**Results:**

At Pmax, CO and CPO were lower in DM compared to obese (CO 16.26 l/min vs. 18.13 l/min, *p* < 0.04; CPO 5.67 W vs. 4.81 W, *p* < 0.01). HE did not differ in CO (18.19 l/min)) or CPO (5.27 W) from OB and DM. Maximum CPO in OB and DM was based on higher stroke volume and blood pressure, while HE had higher heart rates. Pmax was higher (*p* < 0.01) in HE (268 W) compared to OB (108 W) and DM (89 W), mainly caused by a higher (*p* < 0.01) avDO_2_ (HE 18.22 ml/dl, OB 10.45 ml/dl, DM 9.65 ml/dl). Exercise intervention improved Pmax in both groups of patients (+ 16 W in OB, + 12 W in DM), which was attributed to increased avDO_2_, but not to cardiac parameters.

**Conclusions:**

Obese patients had higher cardiac power outputs and were primarily limited by muscular performance, while diabetic patients showed both muscular and cardiac limitations. Healthy subjects had comparable cardiac power outputs with significantly lower pressure-volume loads. Resistance training improved the alteration of our patient groups in exercise capacity. Future research is needed to interpret our findings regarding clinical endpoints, such as mortality and hospitalization.

**Trial registration:**

The study was retrograde registered in the German Clinical Trial Register (DRKS00032545, 24.08.2023).

## Background

Obesity and Diabetes Mellitus Type II (DMTII) are risk factors for cardiovascular diseases and impaired cardiac performance [1–4]. So, patients with this metabolic diseases are declared as patients under risk for heart failure, as the metabolic disease influence cardiac performance and overall outcome, such as mortality and hospitalization [5]. There is ample evidence that exercise capacity measured by maximum oxygen consumption is significantly reduced in obese [6] and DMTII patients [7].

Heart health, cardiac performance capacity and maximum exercise performance are therefore linked in obese and diabetic patients, which is which is reflected, for example, in the term “diabetic heart disease” [4]. The reduction in exercise performance in these patient cohorts can be related to four main factors: cardiac pumping capability, peripheral oxygen extraction, increased arterial blood pressure and respiratory comorbidities, including changes in exercise related respiratory patterns. A reduced cardiac pumping capability leads to a reduced amount of transported oxygen, which directly limits the exercise capacity [8, 9]. The second factor for a limited exercise capacity is an insufficient utilization of the transported oxygen, caused by impaired vascularization, enzymatic and mitochondrial capacity [9]. Hypertensive arterial blood pressure with an increased cardiac afterload is the third factor affecting cardiac output and oxygen consumption and therefore limiting the cardiac pumping capability [10]. Arterial hypertension is a common comorbidity in obese and DMTII patients [11, 12].

In contrast to patients with obesity and DMT II, healthy individuals are mainly limited in their cardiovascular oxygen transport capacity and utilize the majority of the transported oxygen in the working muscles during intense exercises and daily activities [13].

Because obesity and diabetes type II often represent a continuum of disease progression and exercise capacity impairment [2, 7], it is unclear to what extent maximum aerobic exercise capacity is compromised by the main causes mentioned above, cardiac and muscular capacity. It can be assumed, that cardiac pumping capacity decreases in correlation to the disease progress. Comparing patients to a cohort with intact and unbiased regulation, as found in healthy young adults, is appropriate to identify abnormalities in cardiovascular response due to physical stress [14]. It is known that obesity and DMT II induce cardiac regulatory impairments [4, 15].

Direct markers of the left ventricular cardiac pumping capability can be assessed by the cardiac-pressure-volume load during exercise stress tests. In patients with heart failure, detailed cardiac parameters were assessed as indicators of cardiac performance during exercise: cardiac output, stroke work, stroke power output ad cardiac power output [16–18]. Stroke work (SW) is an indicator of pumping capacity per heartbeat, stroke power output (SPO) is a marker of myocardial contractility and wall stress per heartbeat, and the cardiac power output (CPO) as a specific surrogate for overall left ventricular physical cardiac pumping capacity [16, 17]. Age-related reference values of overall exercise performance are known and serve for additional contextualization of the overall exercise capacity in patient cohorts [19–21].

However, in patients with type 2 diabetes and obesity detailed analyses of the mentioned cardiac parameters are rarely reported in studies, especially assessed with cardiopulmonary exercise tests (CPETs).

So, he primary aim of our observational, prospective real-world study was therefore to characterize cardiac capacity (CPO, SW, SPO and CO) and overall exercise capacity in metabolic patient cohorts at risk for heart diseases. To describe exercise performance characteristics more detailed, we analyzed the peripheral aerobic muscular capacity, characterized by the peak power output (Pmax) and the arterio-venous oxygen difference (avDO_2_).Our second aim was to compare the parameters of the primary aim with a healthy reference group without age bias as suggested by Booth and Lees [14], as age is confounder for frailty and cardiovascular diseases itself [22]. The third aspect was to investigate the effects of a standard-of-care hypertrophy and muscular endurance strength training on cardiac and peripheral muscular performance markers in the patients with obesity and DMTII.

We hypothesized that diabetic patients would have reduced exercise capacity based on a reduced cardiac output and a reduced peripheral muscle capacity compared to patients with obesity and to healthy control subjects. Moreover, we assumed a significant correlation of maximal oxygen consumption with avDO_2_, cardiac stroke work and cardiac power output in the patient cohorts and in healthy subjects, respectively.

## Methods

### Subjects

24 patients with obesity, 38 patients with diabetes mellitus type II and 20 healthy subjects were included in the cross-sectional analysis of the present data set. General inclusion criteria were an age above 18 years and the ability to perform light and moderate daily activities. The sub-cohorts were enrolled with an equal distribution of male and female subjects to rule out any gender related bias. All subjects were recruited out of the patients of the sports medicine outpatient clinic of the University of Leipzig.

Inclusion criteria for the two patient subgroups were diagnosed diabetes mellitus type II or obesity with a body mass index above 30 kg/m^2^. Diagnosis of type 2 diabetes mellitus was established by medical records, provided from the patient’s general physician. The clinical status of DMTII in the corresponding patients had to be in stable conditions, regarding no occurrence of hypo- and hyperglycemic events during the observation period and by laboratory values for DMTII (fasting glucose and Hb1ac) within the reference range. Type 2 diabetes mellitus in the group of obese patients was ruled out by laboratory assessments of fasting glucose and HbA1c. The healthy control group included subjects without anamnestic health restrictions and an age range from 18 to 35 years. The amount of sport specific training was limited to less than 4 times a week, to avoid a training status of professional athletes. All participants were informed verbally and in writing prior to the study and provided written informed consent for the study-based data analysis.

### Cross-sectional study part

This study was part of protocol approved by the Medical Ethics Committee of the University of Leipzig in accordance to the Declaration of Helsinki (internal study protocol number 097/17-EK, 089/18-Ek) and was retrograde registered at the German Register for Clinical Trials (24.08.2023; DRKS00032545). Primary endpoint was the total and cardiac -specific exercise capacity, assessed by the maximum oxygen consumption ad cardiac power output.

Participants attended the outpatient clinic twice, on the first examination and diagnostic day and a second consultation day for debriefing and lifestyle instructions. The subjects were instructed to abstain from alcohol and exercise 24 h prior to the first visit. Routine preintervention assessment included a standardized medical history, physical examination, laboratory chemistry, food diary and graded cardiopulmonary exercise test (CPET). The study authors were not involved in the intervention, or routine physician assessments, knowing the inclusion of the data set in this trial. The cardiologist performing the echocardiographic and CPET assessment was not informed of the patient’s enrollment in this specific study and performed the examination as part of usual clinical practice. We will report patient characteristics and physiological variables at rest and at maximal exercise in CPET to investigate maximal cardiac and aerobic muscular capacity. To analyze regulation and find potential dysregulation differentiating the patient group from healthy subjects, we analyzed the cohort at the same external load (watt-match). We determined the external watt-matched load by the maximal load of the diabetic cohort (89 W), resulting in the usage of the load corresponding to 80% in obese and 40% in healthy subjects.

### Longitudinal study part

15 patients with obesity and 18 with diabetes mellitus type 2 were re-examined at the third and fourth visit (second examination and debriefing session) after a standard of care lifestyle intervention in a longitudinal setting according to the pre-intervention assessment procedure. The standard of care intervention consisted of a dietary modification, focusing on nutrient quantity and food quality, combined with muscular strength training (total of 30 sessions) and advice on an active lifestyle to meet typical WHO recommendations for physical activity (150 min per week) [5, 23]. The intervention period had a mean duration of 15 weeks. Regarding this time period, we excluded aging as contributing factor for pre-post assessments. Training was done twice a week and consisted of a warm-up on a cycle ergometer (30–50 W for 10 min), followed by training of six synergistic exercises of the major muscle groups (3 to 5 sets, 10 to 15 repetitions, 50–70% of the weight of the one-repetition-maximum as muscular fatigue was achieved). Repetition ranges were chosen to achieve a hypertrophy and local endurance training effect. The exercises were leg press, latissimus pull-downs, chest press and three individually adapted exercises depending on the individual musculoskeletal symptoms. Weights and resistance were gradually increased during the intervention period.

### Data collection and measurements

**Fig. 1:**
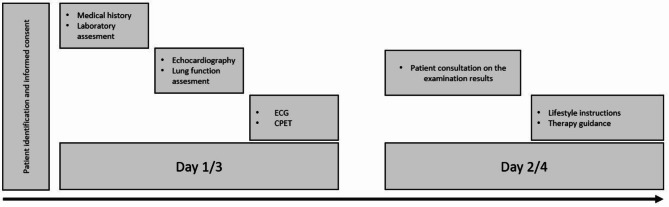
Study Flow Chart. Examination on days 1 and 3, 2 and 4 were identical. 3 Examination days 3 and 4 were only carried out for patients of the longitudinal data analyses in the study cohort. ECG: Electrocardiogram, CPET: Cardiopulmonary exercise test,

Study flow chart is presented in Fig. 1. Pre- and post-intervention examinations were standardized identically. All examinations were carried out and supervised by experienced cardiologists between 10:00 am till 4:00 pm in a clinical laboratory with controlled temperature (21° Celsius). First, medical history, physical examination and echocardiography (Vivid I, GE, USA) were carried out in accordance with the guidelines to rule out higher-grade cardiac diseases [5, 24]. Test of lung function was done to rule out respiratory insufficiencies among the included participants (easy one Pro, NDD, Switzerland). Body composition was assessed via bioimpedance analysis in obese and metabolic patients (medical Bia Corpus RX4000, Germany). Because the healthy subjects did not meet criteria for metabolic diseases, they were not routinely analyzed for body composition in the outpatient clinic. The incremental exercise test was performed according to guidelines for patients presenting a cardiac risk constellation [5, 25]. All data presented were collected before or during the cardiopulmonary exercise test.

The subjects were prepared on a semi-recumbent magnetically-braked bicycle ergometer, and resting data were recorded. The exercise test was then performed at 60 to 75 revolutions per minute, with an initial power of 30 W lasting three minutes and subsequent increments of 10 W per minute until voluntary exhaustion or for clinical reasons, ensuring a minimum CPET time for at least 5 min. The procedure were in line with guidelines of the German and American Guidelines [26, 27] and were published before by our ergometric laboratory [28–30].

Blood pressure (Riva-Rocci), subjective exertion and capillary samples for blood lactate analysis (SuperGL, Dr. Müller, Germany) were measured before, every three minutes during, and at the end of the exercise test. A breath-by-breath spiroergometry (Cosmed k4b2, Italy) were measured continuously after a calibration for each exercise test according to factory specifications. Beat-by-beat impedance cardiography (Manatec Physioflow, France) was carried out at rest and during the exercise tests and the echocardiography at rest was carried out by an experienced cardiologist using GE Vivid I (GE healthcare, Germany).

All collected data were curated by experienced clinicians and academically certified clinical exercise physiologists. Biometrics were monitored by senior medical scientists.

The primary focus in our study was the assessment of cardiac capacity at rest and exercise. We combined echocardiographic and thoracic impedance cardiographic assessment to capitalize on the strengths of both methods [31]. Both methods are capable of measuring the stroke volume by the ventricular outflow or the corresponding aortic flow. The flow-based measurement method corresponding to thoracic impedance cardiography in echocardiography is the stroke volume measurement based on the systolic velocity time integral in the left ventricular outflow tract (VTI) [32]. The echocardiography is well-established as a valid and reliable method to determine the stroke volume at rest [24, 33]. However, during exercise, stress echocardiography is susceptible to artifacts and measurement errors, and requires the specific semi-recumbent and axial left rotated body position. In contrast, impedance cardiography provides a valid method for assessing continuous beat-by-beat hemodynamics at rest and during exercise [34, 35] and is also reliable in terms of sensitivity for changes in cardiac function in patients [36]. Calculating of stroke volume by impedance cardiography based on aortic flow and body surface area may overestimate the stroke volume in subjects with more body fat due to calculating stroke volume based on that higher body surface area. We ran a subgroup analysis to identify or exclude systematic errors. Echocardiographic VTI-based stroke volume measurements were available for in 29 patients of our cohort (15 OB, 14 DMT II).

Respiratory and hemodynamic parameters were averaged over 30 s. Arteriovenous oxygen difference (avDO_2_; ml/dl) was calculated using the Fick equation as avDO_2_ = oxygen consumption/cardiac output assessed by impedance cardiography.

Mean arterial pressure was heart frequency corrected calculated in accordance to the defined correction method of Rogers et Oosthuyse [37]. Stroke work (SW in Newtonmeter) was calculated by stroke volume * mean arterial pressure [38], stroke power output (SPO in Watts) by stroke work/ventricular ejection time and cardiac power output (CPO in Watts) by (stroke work * heart rate) / 60 [39]. Total peripheral resistance (TPR in mmHg*l*min^− 1^) of the arterial blood vessels was calculated by mean arterial pressure/ cardiac output [40]. SW, CPO, SPO and TPR are calculated based on the stroke volumes assessed by impedance cardiography at rest and at maximum load.

### Statistics

All data were checked for plausibility, Gaussian distribution (Shapiro-Wilk normality test), homogeneity of variance (Levene’s test) and statistical outliers (ROUT method). Statistical processing and graph generation were performed using GraphPad Prism 8 (GraphPad Software, USA) and JASP 0.17.1.0 (University of Amsterdam, Netherlands). Analyses of main variables oxygen consumption, cardiac output and arterio-venous oxygen difference were checked for age and sex as a covariate in addition to group comparisons. A priori sample size calculations were performed (G-Power, University of Düsseldorf, Germany) for the primary study aim of cross-sectional between group differences (*n* = 60) and the longitudinal within group differences (*n* = 30) to ensure that minimum required sample sizes were achieved to detect a clinically relevant difference of 10% regarding the maximum oxygen consumption for an average effect size of 0.65 with a power of 0.8. We chose maximum oxygen consumption as a variable for sample size calculation because it is clinically established and for dedicated ventricular variables such as CPO or SW reference values from CPET are rarely reported in obese and diabetic patients. Because cardiac pumping capacity and oxygen uptake are correlated, we used maximum oxygen consumption to plan case numbers. Relevant differences in cardiac output may have been detected when oxygen uptake differed.

Differences between the preintervention cross-sectional analyses (obese vs. DMTII vs. healthy) were tested with an ANOVA followed by post hoc analyses with Fisher’s least significant difference test or with Brown Forsythe test, followed by post hoc analyses with Welches t-tests, if homogeneity of variance was not given (Tables 1, 2 and 3). We conducted a subgroup analysis of stroke volume from thoracic impedance and echocardiography using an intraclass correlation coefficient and a paired samples t-test. There were 29 subjects available for this comparison of stroke volume methods. We performed a post hoc power analysis and determined that the power to detect a clinical difference of 10% in stroke volume was 0.70 at the 0.05 significance level.

Changes attributable to the intervention (Table 4) were examined with a mixed model two-factor ANOVA (time, group, interaction), followed by post-hoc analyses for within group changes with Fisher’s least significance test. Associations were tested for significant correlations (Pearson’s correlation coefficient, significance level *p* < 0.05) and presented as linear regression (r).

## Results

### Cross-sectional study part

#### Resting data and subject characteristics

Resting data from our three subject groups are shown in Table 1. In addition, based on the resting echocardiography we ruled out hemodynamic-relevant valve diseases, heart failure related diastolic compliance issues and hypertrophic myocardial diseases. *P*-values for post-hoc analysis between the patient groups (significance patients) and between HE and the patient groups are presented (significance Healthy vs. patients). Both patient groups differed from the healthy group in most of the parameters we analyzed except for heart rate (HR) and ventricular ejection time (VET). The baseline parameters of our healthy group were in range of young, healthy individuals, without specific adaptions for exercise training. Mean age and diastolic blood pressure differed between obese and DMTII patients and reflected a real-world patient data set. Age and sex did not significantly affect the group comparisons as additional covariate for the main variables of cardiopulmonary exercise capacity VO_2_max, CO and avDO_2_. Age as a confounder in the patient dataset revealed no significant correlation with the maximum power output within patient groups and was therefore excluded from further analysis. As women and men were distributed equally within the cohort, no gender bias affected our data interpretation. Prescription of beta-blockers and anti-hypertensives was tested as a confounder in VO_2_max, CO and avDO_2_, and we found no statistical effect on the results. All patients with arterial hypertension had an already existing specific drug treatment.

### Stroke volume of echocardiography vs. thoracic impedance cardiography

The mean of the echocardiographic stroke volume was 94.2 ml (SD: 17.1 ml) and the mean thoracic impedance cardiography stroke volume measured 96.3 ml (SD: 17.9 ml). We observed good overall agreement of the method with an intraclass correlation coefficient of 0.71 and no significant differences in the means by a paired t-test (*p* > 0.05). Thoracic impedance can thus be considered a valid measure of stroke volume in the underlying data set and confirmed the chosen approach of combining both methods.

**Table 1:** Resting values and patient characteristics

	Obese (numbers/ mean ± SD)	DMT II (numbers/ mean ± SD)	Significance (*p*) Obese - DMTII	Healthy (numbers/ mean ± SD)	Significance (*p*) Healthy- Patients	
sample size (n)	24	38		20		
Age (years)	54.3 ± 13.0	64.3 ± 8.7	*p* < 0.01	26.55 ± 8.5	*p* < 0.01	
Men/women (n)	11/13	18/18		10/10		
Weight	104.8 ± 19.6	99.3 ± 20.5	n.s.	69.6 ± 7.9	*p* < 0.01	
Body Mass Index (kg/m^2^)	36.7 ± 6.9	34.5 ± 5.7	n.s.	22.7 ± 2.1	*p* < 0.01	
RRsys (mmHg)	142.7 ± 15.9	139.9 ± 15.6	n.s.	112.9 ± 10.39	*p* < 0.01	
RRdia (mmHg)	86.7 ± 10.3	80.4 ± 13.0	0.025	74.5 ± 6.3	*p* < 0.01^a^	
arterial hypertension and anti-hypertensive drug prescription (n (%))	15	33		0		
Beta-Blocker prescription	1	6		0		
cardiac output (l/min)	7.72 ± 1.42	7.48 ± 1.41	n.s.	4.91 ± 0.85	*p* < 0.01	
heart rate (bpm)	76.6 ± 12.0	76.9 ± 11.7	n.s.	81.7 ± 15.1	n.s.	
stroke volume (ml)	101.70 ± 17.06	98.76 ± 20.04	n.s.	61.66 ± 13.31	*p* < 0.01	
SW (Nm)	1.43 ± 0.32	1.33 ± 0.33	n.s.	0.76 ± 0.19	*p* < 0.01	
VET (ms)	291 ± 58	310 ± 62	n.s.	289 ± 55	n.s.	
SPO (W)	4.91 (± 1.28	4.40 ± 1.32	n.s.	2.63 ± 0.74	*p* < 0.01	
CPO (W)	1.82 ± 0.46	1.69 ± 0.47	n.s.	1.00 ± 0.20	*p* < 0.01	
TPR (mmHg/l/min)	13.9 ± 2.4	13.8 ± 2.4	n.s.	19.2 ± 3.5	*p* < 0.01	
Smoker/Ex-Smoker (n)	1	8		0		
body fat percentage (%)	39.0 ± 7.40	39.3 ± 7.14		-		

Data presented as mean (standard deviation); RRsys: systolic blood pressure; RRdia: diastolic blood pressure; MAP: mean arterial pressure; HR: heart rate; SV: stroke volume; CO: cardiac output; avDO_2_: arteriovenous oxygen difference; SW: Stroke Work; TPR: total peripheral resistance; VET: left ventricular ejection time; SPO: Stroke power output; CPO: cardiac power output; Significance Patients: *p*-value of differences between the patient groups; Significance Healthy vs. Patients: *p*-value of differences between the reference group and both patient groups (x^a^: differs only from the obese patients)

### Cardiopulmonary exercise test at maximum load

Table 2 shows the physiological parameters in the CPET at maximum load and Table 3 shows the physiological parameters at the same absolute load (Watt match) and, in accordance to Table 1, *p*-values for post-hoc analysis between the patient groups (significance patients) and between HE and the patient groups are presented (significance Healthy vs. patients). At maximum exercise load, obese patients reached higher global performance values (power, oxygen consumption) and higher cardiac parameter values (cardiac output, stroke work, stroke power output, cardiac power output) than diabetic patients.

**Fig. 2:**
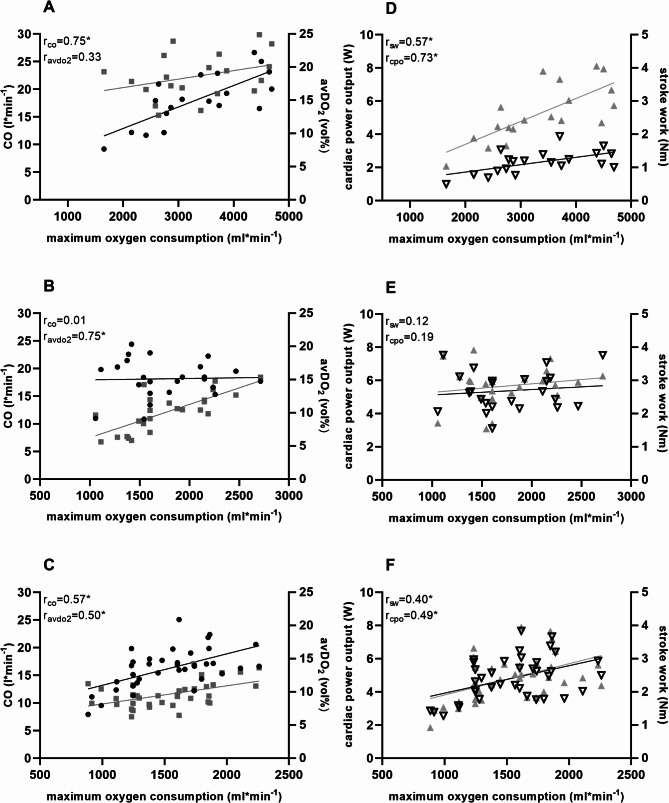
Correlation between maximum oxygen consumption and CO (black dot) and avDO_2_ (gray square) in (**A**) healthy subjects, (**B**) obese patients and (**C**) DMTII patients; correlation between maximum oxygen consumption and stroke work (black empty triangle) and cardiac power output (gray triangle) in (**D**) healthy subjects, (**E**) obese patients and (**F**) DMTII patients, r = correlation coefficient, *= significant correlation

To illustrate the performance structure of these three groups, maximum oxygen consumption related to cardiac output and avDO_2_ are shown in Fig. 2 (A-C). The cardiac load in terms of cardiac power output and stroke work is also shown in Fig. 2 for the study subjects at maximum load in relation to maximum oxygen consumption (D-F). The healthy group (Fig. 2, A) revealed a strong correlation between cardiac output and oxygen consumption (*r* = 0.75, *p* < 0.01) but not with avDO_2_ (*r* = 0.34, *p* = 0.15). Cardiac power output (*r* = 0.74, *p* < 0.01) and stroke work (*r* = 0.57; *p* < 0.01) correlated with increasing aerobic capacity (Fig. 2, D). Obese patients’ maximum oxygen consumption (Fig. 2, B) did not depend on cardiac output (*r* = 0.01, *p* = 0.88), but correlated strongly with avDO_2_ (*r* = 0.75; *p* < 0.01). Their stroke work (*r* = 0.12, *p* = 0.56) and cardiac power output (*r* = 0.19, *p* = 0.37) exhibited no statistically relevant correlations with maximum oxygen consumption (Fig. 2, E).

Our DMTII patients’ maximum oxygen consumption (Fig. 2, C) demonstrated moderate correlative dependence on cardiac output (*r* = 0.57, *p* < 0.01) and avDO_2_ (*r* = 0.50, *p* < 0.01). With increasing maximum oxygen consumption, stroke work (*r* = 0.45, *p* < 0.01) and cardiac power output (*r* = 0.49, *p* < 0.01) also rose in the diabetic subgroup (Fig. 2, F).

**Table 2:** Parameters at maximum load for obese patients, DMTII patients and healthy subjects

	Obese (mean ± SD)	DMT II (mean ± SD)	Significance (*p*) Obese – DMT II	Healthy (mean ± SD)	Significance (*p*) Healthy – patient groups
RRsys (mmHg)	204 ± 24	196 ± 30	n.s.	204 ± 27	n.s.
RRdia (mmHg)	95 ± 13	90 ± 15	n.s.	69 ± 6	*p* < 0.01
MAP (mmHg)	140 ± 14	133 ± 16	n.s.	128 ± 12.5	*p* < 0.01
Power output (W)	108 ± 33	89 ± 24	*p* < 0.01	268 ± 55	*p* < 0.01
Oxygen consumption (ml/min)	1800 ± 421	1543 ± 348	*p* < 0.01	3277 ± 850	*p* < 0.01
Respiratory exchange ratio	0.95 ± 0.12	0.96 ± 0.12	n.s.	1.11 ± 0.09	*p* < 0.01
Lactate (mmol/l)	3.52 ± 1.74	4.07 ± 1.47	n.s.	9.58 ± 1.82	*p* < 0.01
HR (1/min)	129 ± 18	120 ± 15	*p* < 0.02	188 ± 9	*p* < 0.01
SV (ml)	143 ± 27	136 ± 29	n.s.	96 ± 23	*p* < 0.01
CO (l/min)	18.13 ± 3.45	16.26 ± 3.50	*p* < 0.04	18.19 ± 4.61	n.s.
avDO_2_ (ml/dl)	10.45 ± 2.74	9.65 ± 1.89	n.s.	18.22 ± 3.25	*p* < 0.01
SW (Nm)	2.73 ± 0.54	2.40 ± 0.65	*p* < 0.03	1.68 ± 0.49	*p* < 0.01
VET (ms)	217 ± 45	230 ± 46	n.s.	189 ± 25	*p* < 0.01
SPO (W)	13.18 ± 3.01	11.00 ± 4.36	*p* < 0.03	8.90 ± 2.57	*p* < 0.04
CPO (W)	5.67 ± 1.17	4.81 ± 1.37	*p* < 0.01	5.27 ± 1.66	n.s.
TPR (mmHg/l/min)	8.1 ± 1.9	8.5 ± 2.0	n.s.	7.5 ± 1.9	n.s.

Data presented as mean (standard deviation); RRsys: systolic blood pressure; RRdia: diastolic blood pressure; MAP: mean arterial pressure; HR: heart rate; SV: stroke volume; CO: cardiac output; avDO_2_: arteriovenous oxygen difference; SW: Stroke Work; VET: left ventricular ejection time; SPO: Stroke power output; CPO: cardiac power output; TPR: Total peripheral resistance

### Cardiopulmonary exercise test at watt match load

At the same absolute power output (Table 3), our patient groups differed only in their maximum lactate values (*p* < 0.01). The significant group differences we observed are due to the differences between the healthy subjects and two patient groups.

**Table 3:** Parameters at watt-matched load in obese patients, DMTII patients and healthy subjects

	Obese (mean ± SD)	DMTII (mean ± SD)	Healthy (mean ± SD)	Significance ANOVA (*p*)
RRsys (mmHg)	192.0 ± 22.8	196.1 ± 29.7	156.8 ± 18.1	*p* < 0.01
RRdia (mmHg)	93.5 ± 13.5	89.7 ± 15.1	79.1 ± 7.2	*p* < 0.01
MAP (mmHg)	133.8 ± 13.6	132.9 ± 5.8	111.1 ± 10.4	*p* < 0.01
Power output (W)	88.3 ± 29.3	88.6 ± 24.4	89.8 ± 14.8	n.s.
Oxygen consumption (ml/min)	1630 ± 457	1543 ± 348	1566 ± 3 12	n.s.
Ventilation (l/min)	46.55 ± 12.50	48.51 ± 12.36	35.55 ± 7.03	*p* < 0.01
Lactate (mmol/l)	2.42 ± 1.27	4.07 ± 1.47	1.08 ± 0.27	*p* < 0.01
HR (1/min)	119.7 ± 16.1	120.4 ± 15.5	118.8 ± 14.7	n.s.
SV (ml)	136.9 ± 28.7	135.8 ± 29.4	75.7 ± 18.8	*p* < 0.01
CO (l/min)	16.28 ± 3.55	16.26 ± 3.50	8.9 ± 2.0	*p* < 0.01
avDO_2_ (ml/dl)	10.37 ± 3.11	9.65 ± 1.89	18.02 ± 3.26	*p* < 0.01
SW (Nm)	2.45 ± 0.59	2.40 ± 0.65	1.13 ± 0.32	*p* < 0.01
VET (ms)	231.3 ± 50.8	229.5 ± 45.6	242.0 ± 34.5	n.s.
SPO (W)	10.90 ± 2.95	11.00 ± 4.36	4.74 ± 1.30	*p* < 0.01
CPO (W)	4.84 ± 1.13	4.81 ± 1.37	2.22 ± 0.66	*p* < 0.01
TPR (mmHg/l/min)	8.7 ± 2.3	8.5 ± 2.0	12.6 ± 2.	*p* < 0.01

Data presented as mean (standard deviation); RRsys: systolic blood pressure; RRdia: diastolic blood pressure; MAP: mean arterial pressure; HR: heart rate; SV: stroke volume; CO: cardiac output; avDO_2_: arteriovenous oxygen difference; SW: Stroke Work; VET: left ventricular ejection time; SPO: Stroke power output; CPO: cardiac power output; TPR: total peripheral resistance; Significance: post-hoc *p*-value between HE and patient groups

### Longitudinal study part

Our two patient groups’ pre- and post-intervention outcomes are listed in Table 4. As this part focus on the longitudinal interventional outcome, results of the two-way ANOVA were reported as *p*-values for pre vs. post post-hoc test for within groups comparison. We found no significant effect for interaction in our two-way ANOVA and a proportional adaption of both patient groups to the intervention. Thirty-three patient records were included. Both groups of patients increased their maximum aerobic capacity in terms of maximum oxygen consumption and maximum ergometric power output. Pre- and post-interventional CPET result in the same level of exhaustion in both patient groups and between the patient groups, as indicated indirectly by a non-significant difference in the respiratory quotient. The increase in exercise capacity of obese and diabetic patients did not differ significantly (OB: 15.1% vs. DMT II 13.8%). The avDO_2_ rose proportionally with maximum oxygen consumption, while the cardiac performance did not change.

**Table 4:** Pre- and post-intervention parameters under maximum load in obese and DMTII patients

	Obese (numbers or mean ± SD)		Significance (*p*) pre-post OB	DMT II (numbers or mean ± SD)		Significance (*p*) pre-post DM
	pre	post		pre	post	
Men/Women (n)	7/8	7/8		9/9	9/9	
RRsys (mmHg)	202 ± 28	203 ± 22	n.s.	196 ± 29	198 ± 23	n.s.
RRdia (mmHg)	93 ± 11	90 ± 10	n.s.	89 ± 18	86 ± 12	n.s.
MAP (mmHg)	138 ± 14	137 ± 14	n.s.	133 ± 18	131 ± 14	n.s.
Power output (W)	105.3 ± 31.6)	121.3 ± 27.2	*p* < 0.01	88.0 ± 23.8	100.0 ± 29.7	*p* < 0.02
Oxygen consumption (ml/min)	1683 ± 377	1982 ± 380	*p* < 0.01	1523 ± 310	1729 ± 378	*p* < 0.01
Respiratory quotient	0.95 ± 0.12	0.93 ± 0.08	n.s.	0.96 ± (0.12	0.96 (0.08)	n.s.
Lactate (mmol/l)	2.61 ± 1.30	2.73 ± 1.12	n.s.	4.43 ± 1.62	3.98 ± 1.30	n.s.
HR (1/min)	126 ± 15	130 ± 22	n.s.	126 ± 15	124 ± 14	n.s.
SV (ml)	139 ± 25	138 ± 25	n.s.	135 ± 29	141 ± 35	n.s.
CO (l/min)	17.53 ± 3.95	17.76 ± 3.59	n.s.	16.70 ± 2.80	17.43 ± 4.67	n.s.
avDO_2_ (ml/dl)	10.06 ± 2.98	11.43 ± 2.38	0.01	9.23 ± 1.75	10.24 ± 2.26	0.02
SW (Nm)	2.56 ± 0.49	2.52 ± 0.54	n.s.	2.38 ± 0.60	2.44 ± 0.55	n.s.
SPO (W)	12.44 ± 3.80	12.36 ± 3.04	n.s.	11.53 ± 4.75	11.51 ± 5.39	n.s.
CPO (W)	5.39 ± 1.28	5.43 ± 1.35	n.s.	4.95 ± 1.11	5.06 ± 1.32	n.s.

RRsys: systolic blood pressure; RRdia: diastolic blood pressure; MAP: mean arterial pressure; HR: heart rate; SV: stroke volume; CO: cardiac output; avDO_2_: arteriovenous oxygen difference; SW: Stroke Work; SPO: Stroke power output; CPO: cardiac power output

## Discussion

Our research aims and the correspondent hypothesis of reduced cardiac pumping capacity and avDO_2_ in the patient groups was only partially confirmed. At maximal exercise, both patient groups showed reduced maximal oxygen consumption and changes in avDO_2_. Pumping capacity analysis results were heterogeneous. OB showed higher CO and CPO compared to DM. HE showed no differences between the patient groups in these parameters. However, it is interesting to note that the maximum CPO in both patient groups is based on significantly higher stroke volumes and blood pressures at lower maximum heart rates. This results in a higher ventricular load, as indicated by increased stroke work and stroke output.

As expected, maximal oxygen consumption correlated with both CO and avDO_2_ in healthy subjects. In obese patients, this correlation could not be confirmed for the cardiac parameters CO, CPO and SW. Only a correlation between maximal oxygen consumption and avDO_2_ could be demonstrated. In patients with type 2 diabetes, maximal oxygen consumption was correlated with avDO_2_ and cardiac pumping capacity.

The longitudinal analysis of the re-examined patient groups showed that a standard exercise intervention led to adaptations of the peripheral musculature, which subsequently improved maximum oxygen uptake. However, the maximum cardiac pumping capacity did not change.

In the following sections, we discuss our findings in more detail.

### Rest

SW was 88% higher in OB and 75% in DM than in HE according to higher systolic and diastolic blood pressure and higher stroke volumes. Consequently, SPO and CPO were increased as well. When indexed to body weight, the differences in stroke volume between patients and healthy controls were equalized: there was no significant difference between OB (0.97 ml/kg body weight) and DM (0.99 ml/kg) compared to HE (0.89 ml/kg, *p* > 0.05), which in line with other evidence [15].

Body weight adjustment could explain the differences between the groups at rest, but to characterize the true systolic workload the absolute values are more suitable and therefore a body weight adjustment would underestimate the real ventricular stress.

### CPET

Maximum CPO observed in healthy and overweight subjects in our study is consistent with that reported by other studies [41]. To our knowledge, we are the first to demonstrate reduced maximum CPO (-0.86 W, Table 2) in DMTII patients compared to obese patients. The maximum in CPO during CPET in HE resulted from higher heart rates and secondarily from a slightly higher relative rise in stroke volume. Both patient groups increased their stroke volume during CPET by 40%, and HE by 54%. These increases in stroke volume we observed are consistent with other ergometric studies in the elderly [42] and in young healthy individuals [43].

In contrast to the HE group’s regulation, both patient groups presented lower maximum heart rates, but higher blood pressure and stroke volumes, resulting in significantly increased SW and SPO, but not in CPO. The expected maximum heart rate of healthy age-matched individuals in OB was 167 bpm and in DM 162 bpm [19]. Maximum heart rates are therefore 40 bpm (OB; -24%) and 38 bpm (DM; -26%) significantly lower in the patient groups (*p* < 0.01) compared to age-related references. In addition to the direct chronotropic effects of the morbidity, the reduced muscular performance could possibly be a limiting factor for the patient to achieve a higher heart rate. Even if our patient subjects performed the CPET until voluntary exhaustion.

Age- and weight-related maximum power output values were 42% lower in OB (reference: 185 W) and 45% lower in DM (reference: 163 W) compared to reference values (*p* < 0.01), respectively [20].

The reduction in maximum heart rates may cause a compensating by increased stroke volumes to ensure a sufficient cardiac output. This mechanism lead to higher ventricular stress under exercise. Age-related avDO_2_, especially at submaximal loads, should be considered constant in healthy subjects [21]. The reduced avDO_2_ in OB and DM we detected is characteristic of their metabolic disease [44]. Since TPR under maximum exercise load did not differ between OB, DM, and HE, the reduced avDO_2_ cannot be explained by a mismatch in the peripheral blood supply. Reduced capillary density and reduced enzymatic and mitochondrial capacity are the more likely causes [44, 45]. However, focusing on the change in TPR, HE demonstrated significantly larger amplitude than patients from rest to maximum exercise, suggesting superior arterial compliance.

Due to the shift from higher heart rate frequency to pressure-volume load (SW; SPO), maximum CPO did not change in overweight compared to healthy individuals, confirming similar findings by other working groups [39].

Our DMTII patients’ lower SW and CPO values correlated with low maximum oxygen consumption. Their reduced CPO and thus cardiac pumping capacity leads to lower cardiac output, which then exerts a performance-dampening effect in addition to the reduced avDO_2_. This relationship is also known in patients experiencing the genesis of heart failure [17, 18]. Since we found no differences in minute volume of respiration in the watt-matched analysis between the patient groups, breathing patterns seem to explain the differences to a lesser extent. However, compared to healthy individuals, it is apparent that breathing tends to be less efficient.

Taken the findings together, our patient groups required disproportionately more CPO and exploited cardiac output reserve for a smaller increase in absolute power output, resulting in an increase in cardiac load during daily activities and exercise. This was confirmed in the watt-matched data analyses. Here, the same submaximal ergometric loads caused the same cardio-metabolic response in the patient groups. CPO, SW and SPO in healthy subjects were nearly half of the cardiac load in the patient groups (SPO: 10.90 W/ 11.00 W vs. 4.91 W; CPO: 4.84 W/ 4.81 W vs. 2.35 W). OB thus used 85% and DM 100% of their maximum cardiac power output at submaximal watt-controlled loads, whereas healthy subjects only needed 42% of theirs. We can assume that our healthy subjects’ highly efficient muscular oxygen extraction relieves their cardiac load, which is consistent with other studies [45, 46].

The correlation analysis between maximal oxygen consumption, cardiac output, and muscular capacity revealed distinct limitations between groups. In healthy subjects (Fig. 2A), maximal oxygen consumption was primarily limited by maximal cardiac output, as arterial-venous oxygen difference reached near-maximal values, similar to findings in moderately trained young individuals [47]. In contrast, obese patients’ maximal oxygen consumption was mainly limited by avDO_2_, with a lesser contribution from cardiac output (Fig. 2B). Similarly, patients with type 2 diabetes (Fig. 2C) mellitus exhibited a peripheral limitation in avDO_2_ and with reduced cardiac output, which correlated both with lower oxygen consumptions. Both obese and DMT II patients showed reduced aerobic capacity and maximum lactate values, indicating impaired glycolytic function, consistent with findings from Nesti et al. [45] and with morphological cellular alterations in found in muscle cells of patients with heart failure, which also typically showed an impaired muscular oxygen extraction capacity [48, 49]. The capacity to dilate of arterial vessels has also been discussed and demonstrated in patients with heart failure [49] and could also be demonstrated in our cohorts with significantly increased peripheral resistance (Table 2, TPR) in the patient groups. These findings added to the body of the known diabetic heart syndrome, as we found exercise limitations and cardiac capacity alterations that are typical for patients in heart failure patients with preserved ejection fraction [4].

### Exercise training

The 6-month intervention phase, which focused on hypertrophy muscular endurance strength training, resulted in comparable significant improvements in maximum oxygen consumption and maximum power output in both patient groups. This increase in power output was attributable to improvements in peripheral muscle performance as measured by avDO_2_, whereas cardiac output remained unchanged. However, in our patients, the relative cardiac workload at a given exercise intensity decreased by approximately 13% among obese patients and 12% among the diabetics. A 5.4 W CPO in OB before exercise training was associated with 105.3 W before the intervention and 121.3 W after the intervention. In DM, a CPO of 5 W was associated with an 88 W power output before and 100 W after exercise training, respectively.

The corresponding reduction in the rate-pressure product for the same daily load therefore corresponds to less cardiac stress by approximately 12%. Therefore, improved avDO_2_ appears to relieve the heart, especially during submaximal exercise in everyday life.

However, because the CPO in OB and DM patients did not increase, an exercise intervention such as that implemented here did not prove effective in improving cardiac performance. Nonetheless, these findings support current exercise recommendations for patients with DMT II [50], which include strength training. Even after the intervention in the patient groups, only 60% of HE’s average oxygen extraction (avDO_2_) was achieved in the OB group, and 54% in the DM group. Therefore, more intensive endurance training would be necessary to achieve further improvements in avDO_2_. This is certainly where the greatest potential for performance improvement lies, as increasing the stroke volume would demand significantly greater training intensity and frequency. In healthy subjects, e.g., high intensity training [51, 52] or moderate intense constant load trainings would be necessary to enable a volumetric increase in cardiac performance [53]. Patient groups would need to undergo an introduction phase to prepare for such high training volume. During this introduction phase, circulatory performance will be stabilized, while muscular performance increased. Therefore, the intervention demonstrated in this study can be considered a basis for achieving load-tolerant metabolism and musculoskeletal system.

### Limitations

Our patient and reference groups were large enough for this specific experimental setting to achieve the planned effect size. Our findings should be reproduced by investigating lager metabolic patient cohorts in the real-world patient care setting to establish our findings in the clinical setting. Co-morbidities in the patient groups could have influenced outcomes, and should be examined in further studies. In addition, we used a healthy control group, so aged-depended effects should be investigated with an healthy, but age-matched cohort. In addition, studies using bicycle ergometers cannot be generalized to other types of exercise. We analyzed antihypertensive drug treatment as a confounder and found no effect in our data set. However, an effect could not be completely excluded. Especially in larger cohorts, an effect might be more likely to be detected. Regarding our longitudinal setting, a comparison of our strength endurance focused training with an aerobic focused training could have led to different adaptions and may had pronounced cardiac adaptions.

## Conclusion

Detailed evaluation of cardiac power output and pressure-volume stress provided an objective measure of cardiovascular stress at rest and during exercise. A major finding of this study was that cardiac pressure-volume stress and cardiac output are significantly increased at rest and during exercise in patients with obesity and DMTII.

Another key finding was that exercise capacity in obese and diabetic patients was mainly limited by a reduced avDO_2_. This highlights the importance of muscle exercise capacity. Strength training could particularly reverse the avDO_2_-related alteration in exercise capacity.

Future research can observe the stated performance alterations in this cohort in a prospective controlled trial with clinical relevant primary endpoints, such as mortality and hospitalization. Furthermore, we demonstrated reduced cardiac capacity only in obese and diabetic patients. As the risk for heart failure is also increased in other diseases, the observed cardiac parameters should also be investigated in these cohorts.

## Data Availability

The datasets generated during and/or analyzed during the current study are not publicly available due to data protection for pseudonymized data but are partially available for anonymizable data from the corresponding author on reasonable request.

## Abbreviations

ANOVA: One-way analysis of variance
avDO_2_: Arteriovenous oxygen difference
bpm: Beats per minute
CO: Cardiac output
CPET: Cardiopulmonary exercise test
DMTII: Diabetes mellitus type II
DM: Diabetes mellitus type II patient group
ECG: Electrocardiogram
HR: Heart rate
HE: Healthy reference group
J: Joule
MAP: Mean arterial pressure
n.s.: Not significant
n: Number of
OB: Obese patient group
RRdia: Diastolic blood pressure
RRsys: Systolic blood pressure
SD: Standard deviation
SPO: Stroke power output
SV: Stroke volume
SW: Stroke work
TPR: Total peripheral resistance
CPO: Cardiac power output
VO_2_: Oxygen consumption
VTI: Velocity time integral
W: Watt
